# A SNP-mediated lncRNA (*LOC146880*) and microRNA (miR-539-5p) interaction and its potential impact on the NSCLC risk

**DOI:** 10.1186/s13046-020-01652-5

**Published:** 2020-08-14

**Authors:** Tienan Feng, Nannan Feng, Tengteng Zhu, Qiang Li, Qi Zhang, Yu Wang, Ming Gao, Baosen Zhou, Herbert Yu, Min Zheng, Biyun Qian

**Affiliations:** 1 Hongqiao International Institute of Medicine, Shanghai Tongren Hospital/Clinical Research Institute, Shanghai Jiao Tong University School of Medicine, Shanghai, 200025 China; 2 Shanghai Clinical Research Promotion and Development Center, Shanghai Shenkang Hospital Development Center, Shanghai, 200041 China; 3 Key Laboratory of Cancer Prevention and Therapy, Tianjin Medical University Cancer Institute and Hospital, Tianjin, 300060 China; 4 Department of Epidemiology, School of Public Health, China Medical University, Shenyang, 110013 China; 5 Cancer Epidemiology Program, University of Hawaii Cancer Center, 701 Ilalo Street, Honolulu, HI 96813 USA; 6 Tongren Hospital, Shanghai Jiao Tong University School of Medicine, Shanghai, 200336 China; 7 Second Affiliated hospital of Chengdu Medical College, China National Nuclear Corporation 416 Hospital, Chengdu, 610051 Sichuan China

**Keywords:** NSCLC, lncRNA, SNP, miRNA, ENO1

## Abstract

**Background:**

Many cancer-associated single nucleotide polymorphisms (SNPs) are located in the genomic regions of long non-coding RNAs (lncRNAs). Mechanisms of these SNPs in connection to cancer risk are not fully understood.

**Methods:**

Association of SNP (rs140618127) in lncRNA *LOC146880* with non-small cell lung cancer (NSCLC) was evaluated in a case-control study of 2707 individuals. The mechanism of the SNP’s biologic influence was explored with in vitro and in vivo experiments, including plasmid transfection, siRNA knockdown, flow cytometry assessment, and assays of cell proliferation, migration, invasion, and colony formation.

**Results:**

Association analysis showed that A allele of SNP rs140618127 was associated with low risk of NSCLC in the Chinese population. Lab experiments indicated that SNP rs140618127 contained a binding site for miR-539-5p and the binding between miR-539-5p and *LOC146880* resulted in declined phosphorylation of an oncogene, ENO1. The reduced phosphorylation of ENO1 led to decreased phosphorylation of PI3K and Akt, which is further linked to the decline in cell proliferation and tumor progression.

**Conclusion:**

The study demonstrates that SNP rs140618127 in lncRNA loc146880 provides an alternate binding site for microRNA miR-539-5p which affects the phosphorylation of ENO1 and activation of the PI3K and Akt pathway.

## Background

Lung cancer is the most commonly diagnosed cancer (11.6% of the total cases) and the leading cause of cancer death (18.4% of the total cancer deaths) in the world [1]. The majority of lung cancer is non-small cell lung cancer (NSCLC), which accounts for around 85% of all lung cancer cases. Genetic factors may play an important role in an individual’s susceptibility to NSCLC. Long non-coding RNAs (lncRNAs) are a class of non-coding transcripts with 200 nucleotides or more. Increasing evidence suggests that lncRNAs are involved in the occurrence of lung cancer due to their functions as oncogenes or tumor suppressors [2]. Our previous studies indicated that a lncRNA on chromosome 17q24.3, named *LOC146880*, was expressed higher in tumor tissues than in adjacent normal tissues and high expression was associated with poor prognosis of NSCLC [3].

Single-nucleotide polymorphisms (SNPs) in the non-coding regions of the genome have been shown to affect cancer risk via regulating the transcription and/or changing the structure of lncRNA [4–7]. A previous study identified 495,729 SNPs in more than 30,000 human lncRNAs, and a large number of SNPs were predicted to have a potential impact on the microRNA (miRNA)-lncRNA interaction [8]. Here we report the identification of SNP rs140618127 in *LOC146880*, as a new susceptible locus to NSCLC. Bioinformatics analysis predicts that variant rs140618127 (the ‘A’ allele) in *LOC146880* provides an altered secondary structure which may create a binding site for microRNA miR-539-5p [8], sequestering its action on other molecules. Shiraishi et al. conducted a GWAS on lung adenocarcinoma and identified SNP rs7216064 in *BPTF* (17q24.3) in association with the cancer risk (OR = 1.20, *p* = 7e-11) [9]. Seow et al. confirmed that SNP rs7216064 was associated with the risk of lung cancer based on a GWAS study of Asian female non-smokers [10]. We found that SNP rs140618127 was in strong linkage disequilibrium with SNP rs7216064 (LD; r2 > 0.80), and this lncRNA SNP was associated with lung cancer risk (OR = 0.38, *p* = 0.007) in our case-control study of 2707 individuals. To explore the molecular mechanism of SNP rs140618127 in NSCLC development and progression, we evaluated that the biological consequence of *LOC146880* and miR-539-5p interaction, and found that the microRNA behaved like a tumor suppressor [11], which prevented *LOC146880* from interacting with protein ENO1, an oncogene product [12], reducing its phosphorylation. As a result, the phosphorylation of PI3K/AKT was also reduced after the suppression of ENO1 phosphorylation [13], which further inhibited tumor growth and metastasis, leading to a better prognosis of NSCLC.

## Materials and methods

### Study populations

Suspected NSCLC individuals had histopathological or cytologically confirmed diagnosis according to the World Health Organization classification. These study subjects including suspected individuals diagnosed with lung cancer or normal were recruited from the China Medical University (CMU). Distributions of the basic characteristics of the study subjects are provided in Table 1. At recruitment, an informed consent was Illuminated. Only if the subject agreed, he/she was included. This study was approved by the Institutional Review Board at CMU.

**Table 1:** Characteristics of lung cancer patients and healthy controls

Variables	Control	NSCLC	*P* value	OR value	P value	OR value^*^
	*N* = 1509	*N* = 1198				
Age at dx (year)			**< 0.001**		**< 0.001**	
< 60	1203 (79.72%)	604 (50.42%)		1		1
≥ 60	306 (20.28%)	594 (49.58%)		3.87 (3.27–4.58)		3.20 (2.68–3.82)
Gender			**< 0.001**		0.82	
male	422 (27.97%)	544 (45.41%)		1		1
female	1087 (72.03%)	654 (54.59%)		0.47 (0.40–0.55)		0.98 (0.80–1.20)
Smoking status			**< 0.001**		**< 0.001**	
no	1293 (85.69%)	665 (55.60%)		1		1
yes	216 (14.31%)	531 (44.40%)		4.78 (3.98–5.74)		3.89 (3.11–4.87)
SNP rs140618127			**0.007**		**0.018**	
G allele	1476 (97.81%)	1188 (99.17%)		1		1
A allele	33 (2.19%)	10 (0.83%)		0.38 (0.19–0.77)		0.40 (0.18–0.86)

^*^adjusted by smoking/gender/age

### SNP selection and genotyping

SNPs with r^2^ > 0.8 were considered to be in the same LD block. With this criterion, one SNP was selected in each LD block and genotyped using the TaqMan genotyping method in the ABI 7500 Real-Time PCR system (Applied Biosystems). For quality control, we implemented several measures in our genotyping assays, including 1) each plate contained both case and control samples, 2) technicians were blinded to the case/control status of the samples, 3) both positive- and negative-control (no DNA template) samples were included in each 384-well plate, and 4) nearly 8% of the samples were assayed in duplicate and the concordances were between 99.7 and 100%.

### Cell lines

Human NSCLC cell lines (A549 and PC9) and human lung epithelial BEAS-2B cells were purchased from the Cell Bank of Type Culture Collection at the Chinese Academy of Sciences Shanghai Institute of Biochemistry and Cell Biology. These cell lines were passaged for fewer than 6 months. All the cells were tested for mycoplasma and were found to be free from infection. The cells were maintained in DMEM supplemented with 10% FBS and grown without antibiotics in an atmosphere of 5% CO2 and 99% relative humidity at 37 °C.

### 5′ and 3′ RACE and coding prediction of *LOC146880*

We used 5′ and 3′ RACE to determine the transcriptional initiation and termination sites of *LOC146880* with a SMARTe RACE cDNA Amplification kit (Clontech). The Alignment File of a full-length sequence of *LOC146880* obtained from 5′ and 3′ RACE is available upon request.

### Construction of reporter plasmids, transient transfections and luciferase assays

A reporter plasmid in the psiCHECK-2 vector (Promega) was created which contains a 1000-bp *LOC146880* exon region flanking rs140618127 [G] or rs140618127 [A] with the restriction enzymes XhoI and NotI (Fermentas). A549 and PC9 cells were seeded at 1 × 10^5^ cells per well in 24-well plates, and 800 ng of the reporter plasmid and 40 pmol of miR-539-5p mimic (Ambion) were co-transfected into the cells 16 h later using Lipofectamine 2000 (Invitrogen). These cells were collected 24 h after transfection. Renilla luciferase activity was measured and used to normalize the efficiency of transfection.

### RNA extraction and qRT-PCR analysis

Total RNA from the NSCLC tissue specimens and cell lines used in this study was extracted using the TRIzol reagent. First-strand cDNA was synthesized using the SuperScript II reverse transcriptase kit (Invitrogen). Relative RNA levels determined by qPCR were measured on an ABI 7500 sequence detection system (Applied Biosystems) using the SYBR Green method. Βeta-actin was employed as an internal control for the quantification of *LOC146880* and the mRNA levels of other genes. For miRNA quantification, small nuclear RNA U6 was used as an endogenous control. The relative expression of RNA was calculated using the comparative Ct method.

### Subcellular fractionation

Cytosolic and nuclear fractions of A549 and BEAS-2B cells were prepared and collected according to the instructions of the Nuclear/Cytoplasmic Isolation kit (Biovision). *LOC146880* was mainly detected in the nuclear fraction, although it was also present in the cytoplasm (Fig. S1).

### RNA pulldown and mass spectrometry analysis

RNA pulldown assays were performed following the protocol described below. Briefly, biotinylated *LOC146880* or antisense *LOC146880* was incubated with cellular protein extracts from A549 cells, and streptavidin beads were then added. Recovered proteins associated with *LOC146880* or antisense *LOC146880* were excised, and proteomics screening was accomplished by mass spectrometry analysis on a MALDI-TOF instrument (Bruker Daltonics). In vitro transcription of *LOC146880* and its deletion fragments were analyzed with primers containing the T7 promoter sequence.

### RNA immunoprecipitation assays

RIP experiments were performed using the Magna RIP RNA-Binding Protein Immunoprecipitation kit (Millipore). Antibodies against ENO1 (Abcam) or control proteins were diluted at 1:50. Total RNA (input control) and precipitation with the isotype control (IgG) for each antibody were assayed simultaneously. The co-precipitated RNAs were detected by RT-qPCR.

### Plasmid construction and transfection

To construct a lentiviral vector expressing human *LOC146880* (NR_026899), a full-length of *LOC146880* cDNA containing rs140618127 [G] or rs140618127 [A] was commercially synthesized (GeneChem) and subcloned into the AgeI and NheI sites of the GV367-IRES-Puromycin lentiviral expression vector (GeneChem). To produce lentivirus containing *LOC146880*, 293 T cells were cotransfected with the vector described above and lentiviral vector packaging system (GeneChem) using Lipofectamine 2000. Infectious lentiviruses were collected at 48 h after transfection and filtered through 0.45-μm PVDF filters for analysis of genotype. After conformation, these lentiviruses were designated to *LOC146880* [G] or *LOC146880* [A]. We used the GV367-IRES-Puromycin empty vector as a negative control. The virus-containing pellet was dissolved in DMEM, and aliquots of the solution were stored at − 80 °C. A549 and PC9 cells were infected with concentrated virus in the presence of polybrene (Sigma-Aldrich). The supernatant was replaced with complete culture medium after 24 h, followed by selection with puromycin, and the expression of *LOC146880* in infected cells was verified by qPCR.

### Cell lysis and immunoprecipitation

Cells were homogenized in 1× RIPA buffer supplemented with Protease/Phosphatase Inhibitor Cocktail (Pierce). Cell lysates were centrifuged, and the supernatants were prepared for immunoblotting or immunoprecipitation with the antibodies described below. Immunoblot signal was detected using Clarity Western ECL Substrate (Thermo Fisher).

### Immunoblot assays

Protein extracts from cells or immunoprecipitation samples were prepared using detergent-containing lysis buffer. Total protein (60 μg) was subjected to SDS-PAGE and transferred to PVDF membrane (Millipore). Antibodies against ENO1 (Abcam, ab155102), ENO1 phosphorylated at C-terminal inhibitory site Tyr44 (StressMarq Bioscience, spc-965D), PI3K (CST, 13666S), PI3K phosphorylated at Tyr458 (CST, 4228S), AKT (CST, 2938S), AKT phosphorylated at Ser473 (CST, 9018S), PCNA (CST, 2586S), NF-kB (CST, 8242S), Vimentin (Abcam, ab92547), β-Catenin (CST, 8480S), E-cadherin (CST, 14472S), N-Cadherin (Abcam, ab18203), and β-Actin (Sigma-Aldrich, A1978-200UL) were used. Membranes were incubated overnight at 4 °C with primary antibody diluted 1:1000, and proteins were detected with the Odyssey near infrared dual-color laser imaging system (LICOR).

### Analysis of cell proliferation, migration, invasion, cell cycle, and colony formation

Cells were seeded in 96-well flat-bottom plates, with 2000 cells in 100 μl cell suspension in each well. After culture, cell viability was measured with the CCK-8 assay. Each experiment with six replicates was repeated three times. For cell cycle analysis, cells were collected and fixed in 70% ethanol overnight at 4 °C. Single-cell suspensions were labeled with 50 μg/ml Propidium Iodide (Sigma) and analyzed by flow cytometry (Beckman Coulter). For colony formation, 2000 cells were seeded in 65-mm culture dishes and allowed to grow until visible colonies formed in complete growth medium (2 weeks). Cell colonies were fixed with methanol, stained with crystal violet and counted. Invasion assays were performed in Millicell chambers in triplicate. The 8-μm pore inserts were coated with 30 μg of Matrigel (BD Biosciences). Cells (5 × 10^4^) were added to the coated filters in serum-free medium. PMI-1640 medium containing 20% FBS was filled in the lower chambers as a chemo attractant. After 24 h at 37 °C in an incubator supplied with 5% CO^2^, cells that migrated through the filters were fixed with methanol and stained with crystal violet. Cell numbers in three random fields were counted. The migration assay was conducted in a similar fashion without coating the filters with Matrigel.

### Experiments on xenograft animals

Ten male BALB/c mice (5 weeks old) were kept in a specific pathogen-free grade environment. All animal experiments were approved by the Animal Care and Use Committee of Shanghai Jiao Tong University School of Medicine (Shanghai, China). All applicable guidelines of the Animal Care and Use Committee of Shanghai Jiao Tong University School of Medicine for the care and use of animals were followed. PC9 cells of rs140618127 [A] and rs140618127 [G] type were collected and resuspended in PBS at a concentration of 1 × 10^8^ cells/mL and mixed with Matrigel® at a ratio of 1:1 respectively. The mixture (0.1 mL) was subcutaneously injected into two sides of the hind flank regions of the mice, rs140618127 [A] and rs140618127 [G] cells in the same mouse. Tumor size was measured once every 2 days using a Vernier caliper across its two perpendicular diameters, and tumor volume was calculated using the following formula: V = 1/2*a*b^2^; where V is the tumor volume, a is the largest diameter, and b is the smallest diameter. After 4 weeks of treatment, all mice were sacrificed, and their tumors were collected and weighed. Histological evaluation of the tumor samples was performed.

### Histopathological analyses

Tumor tissues from the animals were fixed in 4% paraformaldehyde (BOSTER, Wuhan, China) for 48 h at room temperature. The fixed tissues were then dehydrated in a graded series of alcohol, cleaned in xylene, and embedded in paraffin. A rotary microtome was used to section paraffin the blocks into 4-μm thick sections. The sections were deparaffinized and stained with hematoxylin and eosin (H&E). A light microscope (Olympus) was used to examine the stained tissue sections.

### Statistical analysis

The association between SNP rs140618127 and NSCLC risk was analyzed under an additive model using the unconditional logistic regression model adjusted for age, sex, and smoking status. Results of laboratory experiments were presented as Means ± SD. Student’s t test was used to compare means between two groups, and ANOVA was employed for comparison of more than two groups. Repeated ANOVA was employed for comparison of more than two groups which contained repeated measure data. All the statistical analyses were performed using Statistical Product and Service Solutions (SPSS) software (version 19.0) and GraphPad Prism Version 8.0 (GraphPad Software, San Diego CA, USA).

## Results

### SNP rs140618127 (G > A) in *LOC146880* and NSCLC risk

SNP in *LOC146880* (rs140618127) is in strong linkage disequilibrium with SNP rs7216064 (r^2^ > 0.80) which is a GWAS-discovered risk allele for NSCLC. We found that SNP rs140618127 (G > A) in the exon of *LOC146880* (chr17: 64758273) was associated with the risk of NSCLC; the ‘A’ allele, compared to ‘G’, had an adjusted odds ratio (OR) 0.40 (0.18–0.86) in a case-control study of 2707 subjects (Table 1). Stratified analyses suggested that this effect was more evidence in those who were ≥ 60 years old, female, and non-smokers (Supplemental Table S1). The minor allele frequency of SNP rs140618127 is low globally, < 1%, but can be high as 20% in some American populations (see Suppl. 1).

### Effects of *LOC146880* with rs140618127 [a] on cell proliferation and behaviors

**Fig. 1:**
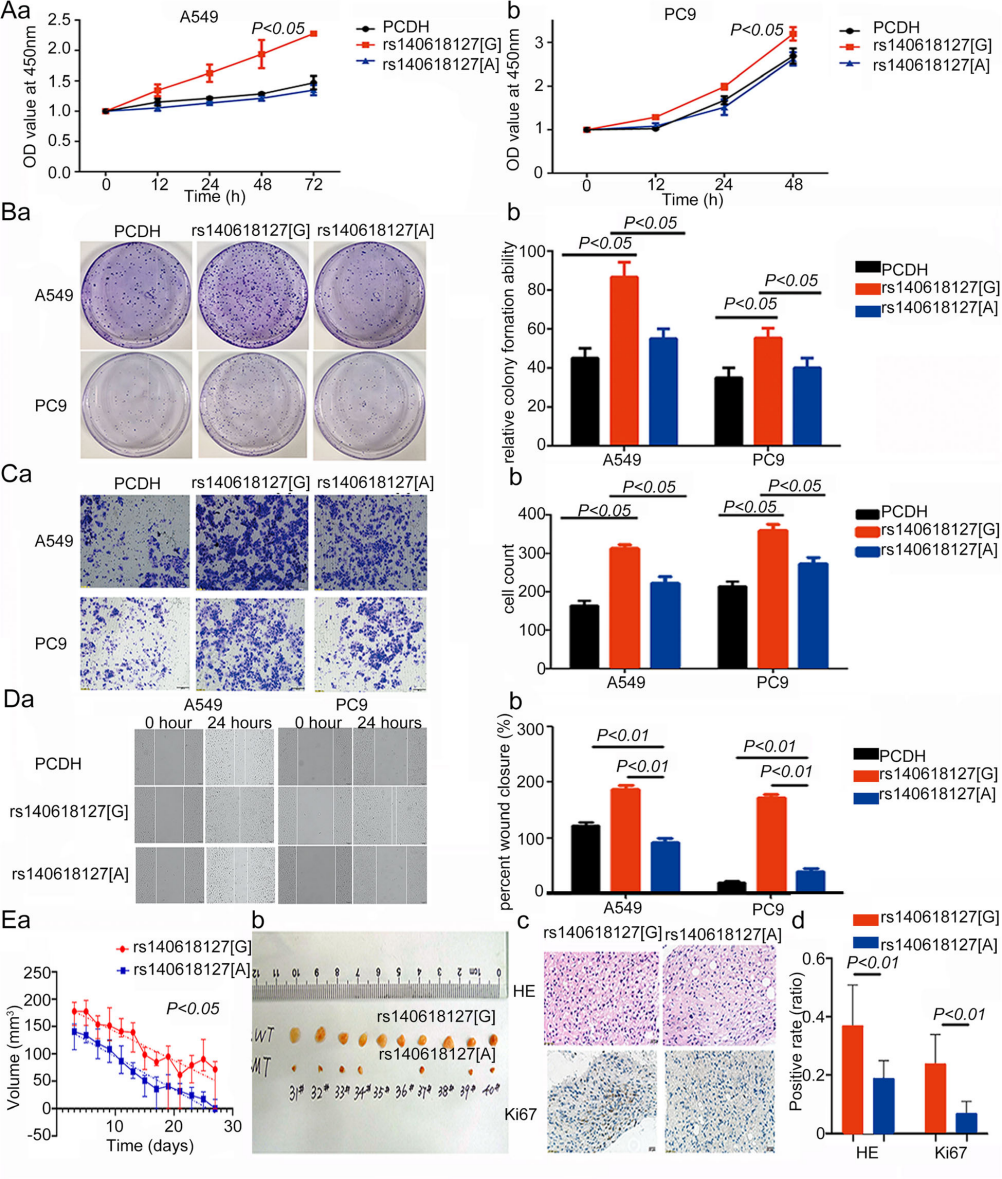
rs140618127[A] inhibits NSCLC cell proliferation and EMT process: (A) proliferation assay of different samples which were compared by repeated ANOVA; (B) clone formation ability of different samples which were compared by ANOVA; (C) wound healing assay of different samples which were compared by ANOVA; (D) cell invasion assay of different samples which were compared by ANOVA; (e) cancer protective effect of rs140618127[A] in xenograft animals which were illuminated by repeated ANOVA and t-test

We examined the effects of *LOC146880* on cell proliferation by its allele at rs140618127, and found that overexpression of rs140618127 [A] in the NSCLC cell lines A549 and PC9 (both with the G allele at rs140618127) substantially reduced the rate of cell proliferation when compared with rs140618127 [G] (Fig. 1A). Colony formation ability in both A549 and PC9 cells was markedly suppressed by rs140618127 [A] when compared with rs140618127 [G] (Fig. 1B). Overexpression of rs140618127 [A] significantly suppressed the invasion and migration of NSCLC cells (Fig. 1C & 1D). Tumor size in a xenograft animal model of PC9 was decreased in both genotype groups, but the decline in tumor size was greater for rs140618127 [A] than for rs140618127 [G] (*P* < 0.05). There was no significant difference in tumor size between the vector control group and wild type, rs140618127 [G] (Fig. S2). H&E staining showed that tumors of rs140618127 [A] possessed less malignant morphology (Fig. 1E). Together, these results indicate that rs140618127 [A] can inhibit the growth of lung cancer more in vitro and in vivo compared to rs140618127 [G].

### Interaction between *LOC146880* and miR-539-5p

**Fig. 2:**
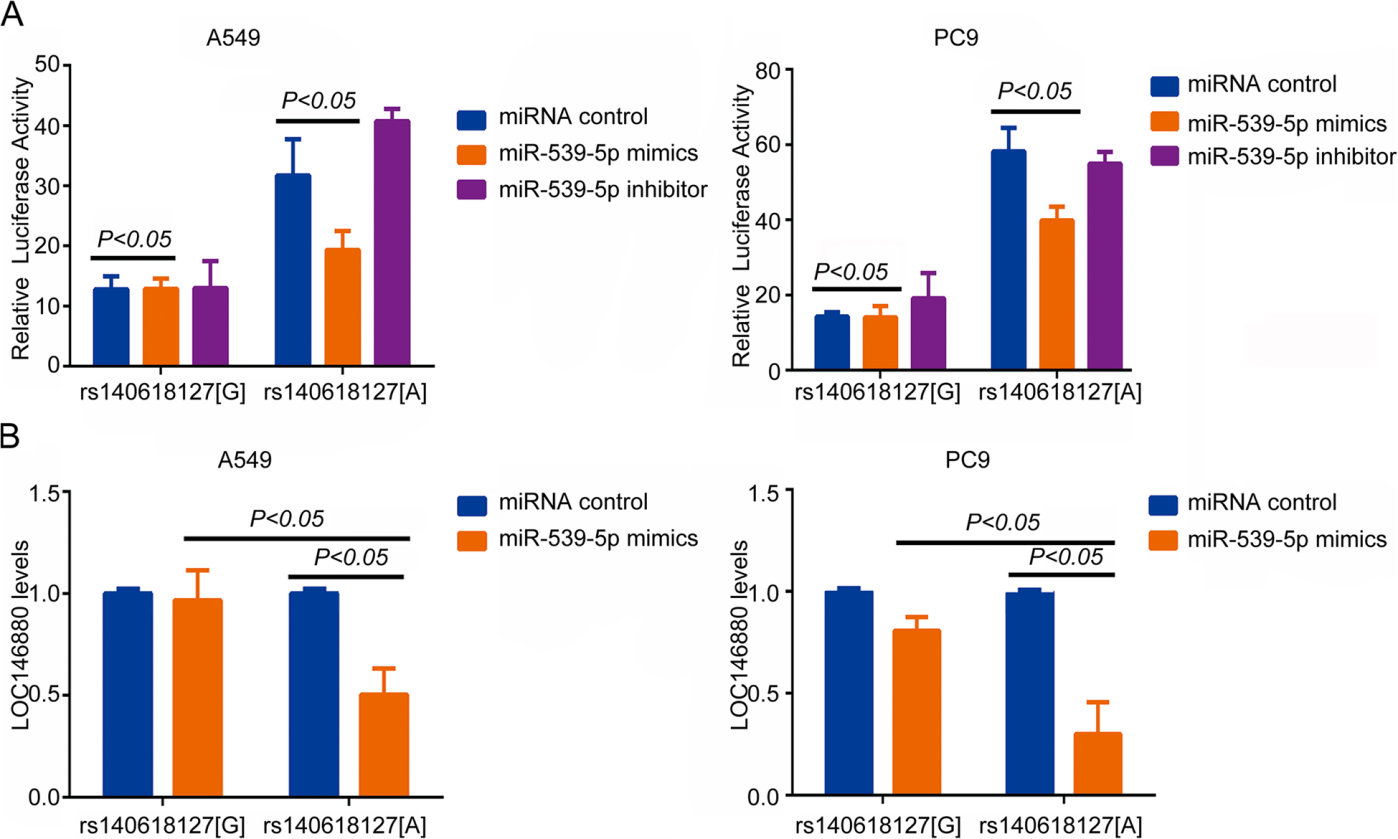
rs14061812[G]/[A] expression of different condition in A549 and PC cell lines: (a) luciferase activity of different conditions which were illuminated by ANOVA; (b) *LOC146880* expression level of different conditions which were compared by t-test

Evidence suggests that SNPs in lncRNAs may generate new interacting sites between lncRNAs and other transcripts, such as miRNAs [14]. Using an online software lncRNASNP (http://bioinfo.life.hust.edu.cn/lncRNASNP) [15], we found that several SNPs in *LOC146880* were predicted to have such a possibility and SNP rs140618127 was indicated to lie within a putative binding site for miR-539-5p. The G > A mutation at rs140618127 was predicted to change the local folding structures and free energy of *LOC146880* which might create a binding site for miR-539-5p. Following this prediction, we investigated whether miR-539-5p interacts with *LOC146880* based on its genotype at rs140618127. Luciferase reporter assays showed that, in comparison to the construct containing rs140618127 [G], the construct with the ‘A’ allele had significantly reduced luciferase activity in the presence of miR-539-5p, suggesting more interaction of miR-539-5p with *LOC146880* [A] than with *LOC146880* [G] (Fig. 2A). The interaction between miR-539-5p and *LOC146880* [A] could be blocked by the miR-539-5p inhibitor; miR-539-5p is constitutively expressed in both A549 and PC9 cells. In cells stably overexpressing *LOC146880*, miR-539-5p only decreased the levels of *LOC146880* with rs140618127 [A], not allele G, indicating that allele A is a target of miR-539-5p (Fig. 2B).

### Interaction between *LOC146880* and ENO1

**Fig. 3:**
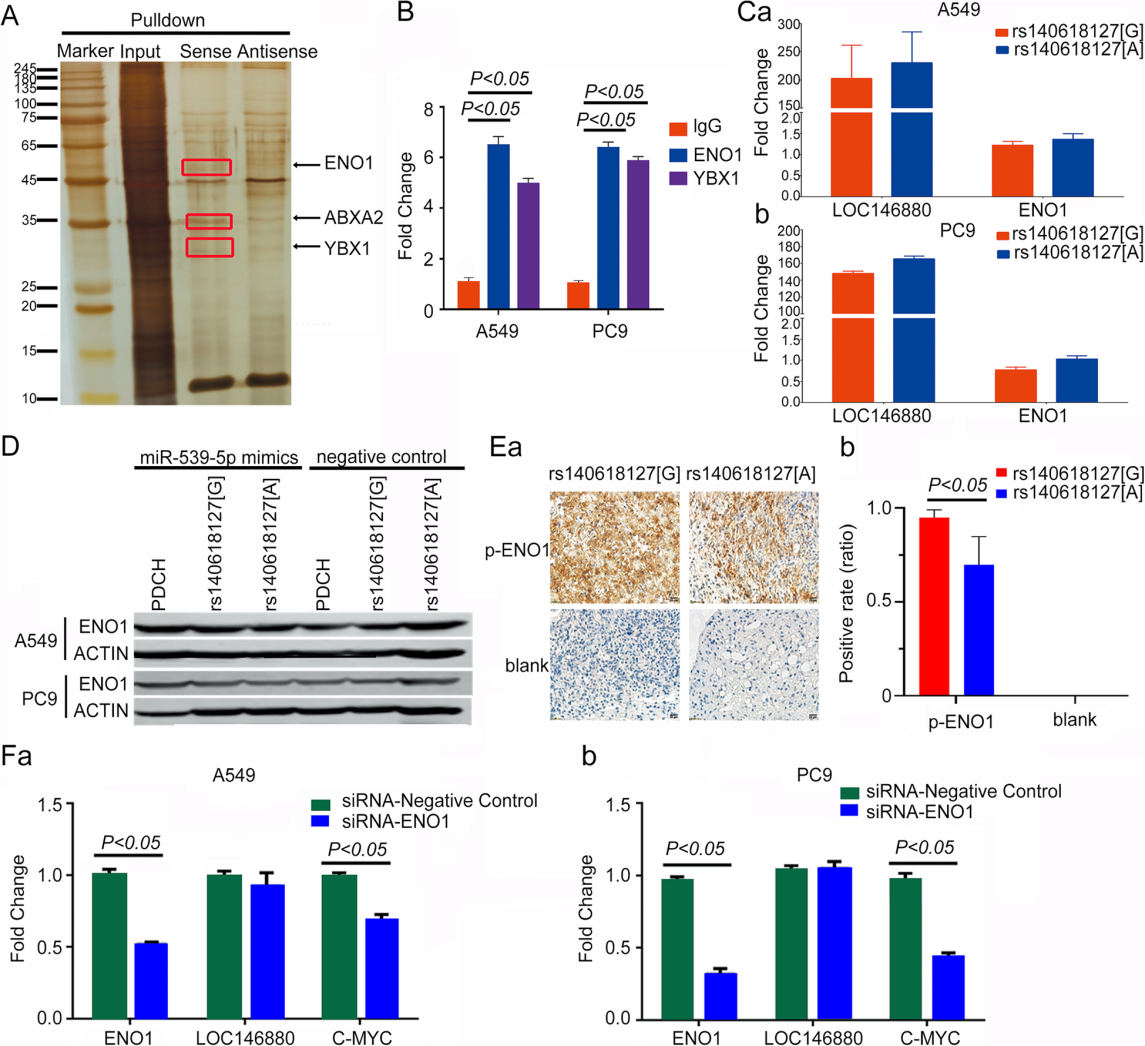
*LOC146880* promotes ENO1 activation in a variant-specific manner: (A) pulldown assay-mass spectrum of identification-silver staining; (B) RIP assay with A549 and PC9 cell lines which were compared by ANOVA; (C, D) ENO1 mRNA expression and protein level of rs140618127[G]/[A] by overexpression plasmid transfection which were compared by t-test; (E) H&E staining of ENO1 phosphorylation in vivo which were compared by t-test; (F) ENO1, *LOC146880* and c-MYC expression level after ENO1-siRNA transfection which were compared by t-test

Using the RNA pulldown assay, we isolated a *LOC146880* with rs140618127[G]-protein complex. Mass spectrometry analysis showed that there were three proteins in this complex and the most abundant one (compared to anti-sense one) was ENO1 (Fig. 3A). We then selected ENO1 for validation, detecting ENO1 in three independent RNA pulldown assays. RNA immunoprecipitation (RIP) assays also showed enrichment of *LOC146880* in the complexes precipitated with ENO1 antibody as compared with IgG or another irrelevant antibody, indicating that ENO1 may be a key target protein of *LOC146880* (Fig. 3b). Next, we evaluated the consequences of the interaction between *LOC146880* and ENO1. We found that ENO1 mRNA expression and protein level were not significantly different (*P* > 0.05, see Fig. S3) in the cells overexpressing *LOC146880* with rs140618127[A] or rs140618127[G] in the presence of miR-539-5p (Fig. 3C & 3D. However, H&E staining of xenograft tumors in mice showed that phosphorylated ENO1 was higher in rs140618127 [G] than in [A] (Fig. 3E). The expression of C-MYC, a downstream target of ENO1, was decreased remarkably when the cells were transfected with a siRNA against ENO1 (Fig. 3F).

### Regulation of PI3K/AKT signal by *LOC146880* via ENO1 phosphorylation

**Fig. 4:**
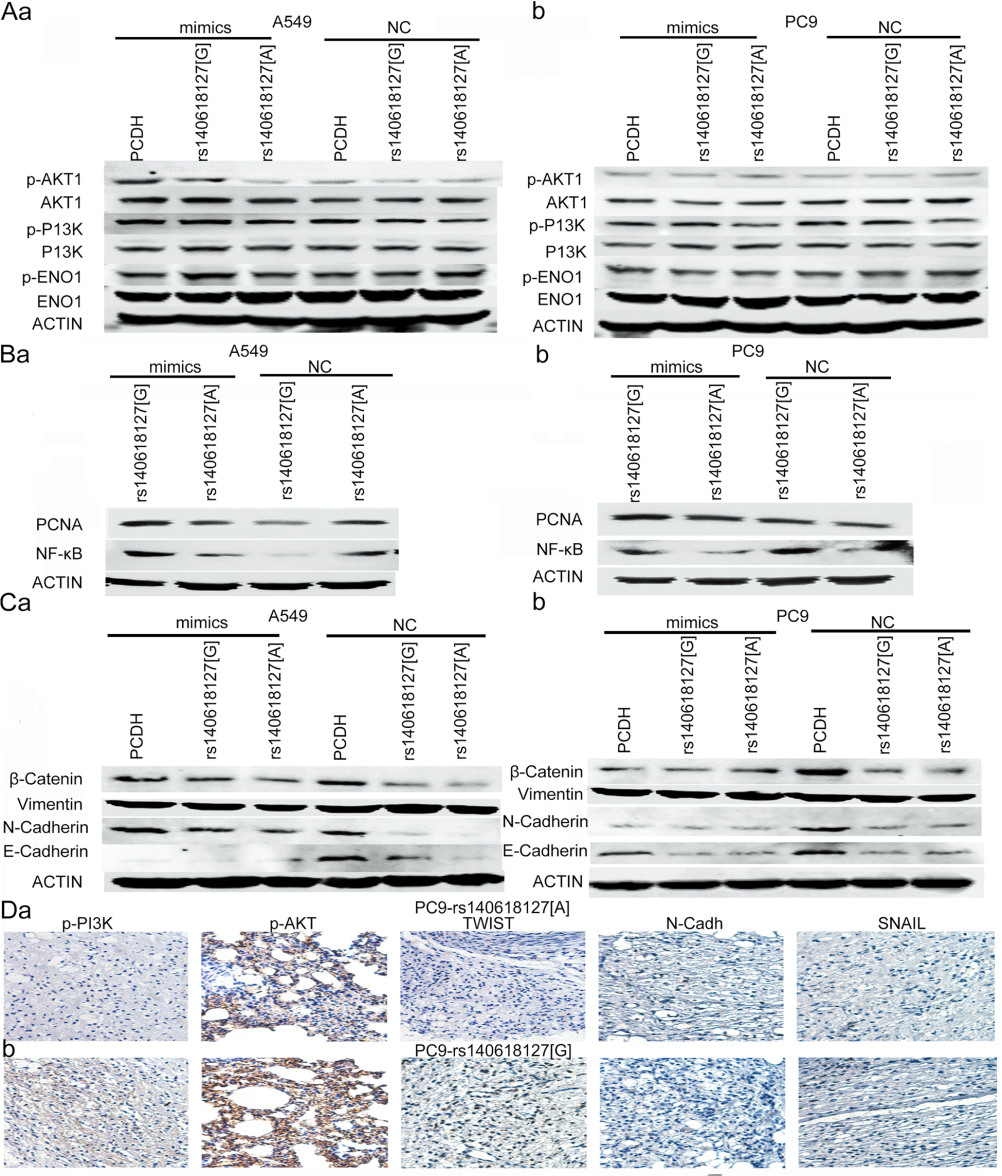
*LOC146880* regulates PI3K/AKT signaling via ENO1 (A549 & PC9): (A) ENO1/p-ENO1, PI3K/p-PI3K, AKT/p-AKT protein level using rs140618127[G]/[A] overexpression plasmid transfection; (B) PCNA, NF-kB protein level using rs140618127[G]/[A] overexpression plasmid transfection; (C) β-Catenin, Vimentin, N-Cadherin, E-Cadherin protein level using rs140618127[G]/[A] overexpression plasmid transfection; (D) H&E staining of p-PI3K, p-Akt, TWIST, N-Cadhersin and SNAIL

We found that ENO1 phosphorylation was markedly decreased in cells overexpressing rs140618127 [A] as compared with those overexpressing rs140618127 [G] in the presence of miR-539-5p mimics (Fig. 4A, Fig. S4, and Fig. S5). Using in-silico prediction tools [16, 17], we identified a phosphorylation site at Tyr44 in the protein based on the PDB database (Fig. S5) [18]. We next investigated the impact of altered *LOC146880* levels on the downstream signal of ENO1. Since our results described above indicated that *LOC146880* overexpression increased cell proliferation, migration, and invasion, we focused our investigation on the PI3K/AKT-NF-kB signaling. The total amount of PI3K and AKT proteins was not significantly different between cells overexpressing rs140618127 [A] and [G]. However, we observed that protein phosphorylation levels affected the expression of downstream molecules in the PI3K/AKT signaling in A549 (Fig. S4). Cells overexpressing *LOC146880* with rs140618127 [A], showed substantial decreases in NF-kB, PCNA, Vemintin, and N-cadherin levels while their β-catenin and E-cadherin levels were significantly increased when compared with the same cells overexpressing *LOC146880* with rs140618127 [G] (Fig. 4B, Fig. 4C, Fig. S7, and Fig. S8). Immunohistochemical staining of xenograft tumors showed that p-PI3K, p-AKT, TWIST, N-Cadh, and SNAIL were all significantly higher in rs140618127 [G] than in [A] (Fig. 4D and Fig. S9).

## Discussion

**Fig. 5:**
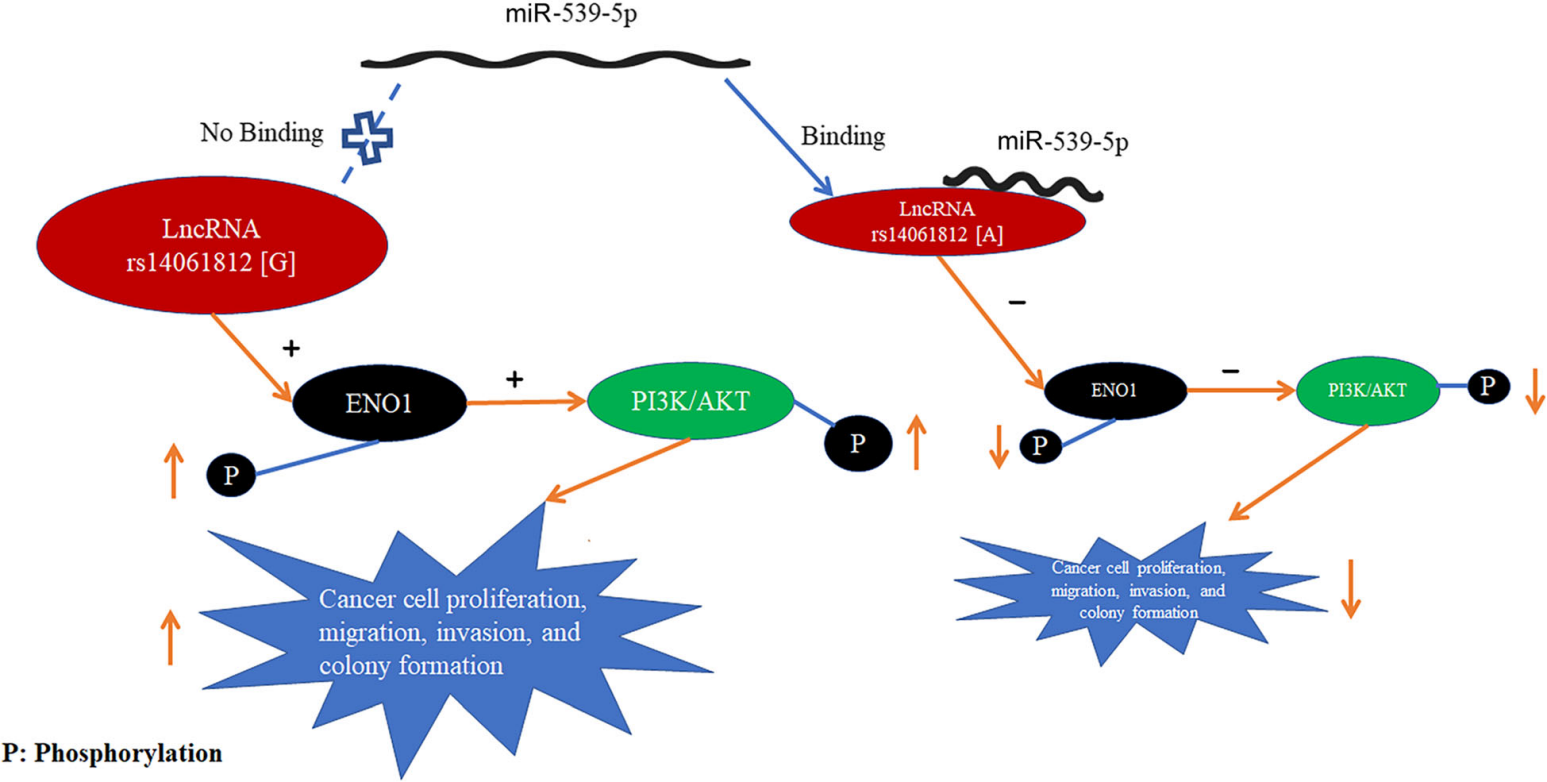
Diagrammatic sketch of rs14061812-mediated NSCLC tumorigenesis: *LOC146880* (rs14061812[G]) increases ENO1’s phosphorylation, resulting in activating PI3K/AKT signaling pathway and NSCLC tumorigenesis while *LOC146880*(rs14061812[A]) binds to miR-539-5p, decreases ENO1’s phosphorylation, resulting in deactivating PI3K/AKT signaling pathway and NSCLC tumorigenesis

In this study, we found that SNP rs140618127 in *LOC144680* contained a binding site for miR-539-5p, and the binding between miR-539-5p and *LOC146880* resulted in declined phosphorylation of an oncogene, ENO1, which was found to be a downstream target of *LOC146880*. Furthermore, the reduced phosphorylation of ENO1 led to decreased phosphorylation of PI3K and Akt, which was linked to the decline in tumor cell proliferation and progress. The entire process of how SNP rs140618127 influences NSCLC is depicted in Fig. 5. Our case-control study supports the notion that SNP rs140618127 genotype [A] may have a protective effect on NSCLC compared to genotype [G]. In a previous study, we found that *LOC146880* expression was significantly higher in NSCLC tumors than adjacent normal tissues, suggesting a possible oncogenic role for *LOC146880* [3].

There has been an increasing interest in understanding the mechanisms of rare genetic variants in lncRNAs in relation to the complex traits and diseases [19, 20]. Ingle et al. suggested that genetic polymorphisms in lncRNA *MIR2052HG* offer a pharmacogenomic basis for the response of breast cancer patients to aromatase inhibitor therapy [21]. Tang et al. indicated that SNP rs9839776 in *SOX2OT* was significantly associated with breast cancer possibly via influencing the expression of *SOX2OT* [22]. Redis et al. demonstrated that the GWAS-identified SNP rs6983267 on 8q24 is in a lncRNA gene called *CCAT2* which regulates cancer cell metabolism in an allele-specific manner through binding to the cleavage factor I complex. This complex is implicated in an allele-specific regulatory mechanism of cancer metabolism orchestrated by alleles of the lncRNA [22, 23]. Russell et al. identified a neuroblastoma susceptibility locus rs9295534 located in the upstream enhancer of a tumor suppressor CASC15-S. The SNP could decrease the transcriptional activity of CASC15-S and be associated with the disease outcome [24]. Wang et al. found that SNP rs965513, a locus on 9q22 in the *FOXE1* gene and *lnc-PTCSC2*, was associated with the risk of papillary thyroid carcinoma [25].

In this study, we found that a lncRNA could regulate the function of a protein via its phosphorylation, with little influence on gene expression or protein concentration. Similar findings have been reported before in which the phosphorylation site of a protein can be blocked by a lncRNA leading to decreased phosphorylation. For example, NF-kB can be inhibited by a long noncoding RNA which directly blocks IKB phosphorylation in breast cancer [26]. LncRNA can also bind to proteins, increasing or decreasing their phosphorylation via another protein. LncRNA *DANCR* and *PANDAR* influence the phosphorylation of serine in RXRA and SFRS2 via GSK3β and P53 in breast and ovarian, respectively [27, 28]. GSK3β’s phosphorylation in breast cancer was reported to be reduced by lncRNA *NLIPMT* [29]. The phosphorylation of ULK1 can be suppressed by lncRNA *HOTAIR* in NSCLC [30]. *LINC00675* enhances the phosphorylation of vimentin on Ser83 to suppress gastric cancer progression [31]. Our finding of a lncRNA’s impact on the phosphorylation of a protein was quite unique and interesting because it is achieved by a microRNA through a polymorphic site in *LOC146880*.

In our study, we found that a G to A transition at rs140618127 in *LOC146880* could turn into a binding site for a microRNA and miR-539-5p was indeed the target. Interestingly, the wildtype of *LOC146880* had no interaction with the microRNA at all. This SNP has not been reported before in any studies [32]. However, miR-539 is known to be a tumor suppressor [33, 34]. Our finding of miR-539’s binding to loc146880 provided new insights into a possible mechanism that explains the biologic function of miR-539 as a tumor suppressor. This effect takes place when a microRNA and lncRNA interact through a polymorphic site which results in changes in phosphorylation in a protein ENO1 that the lncRNA may target on. Low levels of *LOC146880* did not influence the mRNA expression or protein levels of ENO1, but suppressed the phosphorylation of ENO1. ENO1 is a metabolic enzyme involved in the synthesis of pyruvate. It also acts as a plasminogen receptor and mediates the activation of plasmin and extracellular matrix degradation. In tumor cells, ENO1 is up-regulated and supports the Warburg effect. The protein is located on the cell surface where it promotes cancer invasion, and is subjected to substantial post-translational modifications, namely acetylation, methylation, and phosphorylation [35]. Reduced phosphorylation of ENO1 lowers the PI3K/Akt signal, which results in slower cell migration or invasion of NSCLC.

The SNP-based interaction between miRNA and lncRNA in regulation of protein function has been hypothesized and predicted by Ya-Ru et al. but few studied have provided evidence [15]. Our study was the first to show the interaction between *LOC146880* and mir-539-5p in the NSCLC and to elucidate the downstream mechanism involving tumor growth and metastasis. The modulation model of the lncRNA and miRNA is not the classical competing endogenous RNAs (ceRNA). How *LOC146880* interacts with ENO1 to regulate its phosphorylation and downstream signals remains to be elucidated. Although the ‘A’ allele of rs140618127 is low in general, some racial groups still have a relatively high frequency. In some Caucasian populations, the ‘A’ allele frequency is close to 20%.

## Conclusions

We found in a case-control study of 2706 Chinese that SNP rs140618127 in *LOC146880* was associated with the risk of NSCLC. People with the G allele of rs140618127 had higher risk than those with the A allele. Our in vitro and in vivo experiments demonstrated that *LOC146880* was an oncogene and the G allele of rs140618127 had stronger oncogenic effects on lung cancer cells than the A allele in *LOC146880*. This differential effect appeared to come from the binding of a microRNA, miR-539, to the A allele, but not the G allele at rs140618127. The microRNA binding prevented the lncRNA’s interaction with its downstream target ENO1, which led to the reduction of ENO1 phosphorylation and suppression of the PI3K/AKT signaling, resulting in lower tumor cell proliferation and less aggressive cell behaviors.

## Supplementary information

**Additional file 1 Fig. S1.** Locations of *LOC146880* (A549 & BEAS2B): *LOC146880* locals mainly in cytoplasm. The comparison between two groups using t-test.

**Additional file 2 Fig. S2.** Comparison of tumor size between vector control group and the wide type: There was no significant difference of tumor size between vector control group and the wide type, rs14061812[G]

**Additional file 3 Fig. S3.** Comparison of ENO1 protein level of rs140618127[G]/[A] by overexpression plasmid transfection which were compared by t-test.

**Additional file 4 Fig. S4.** Comparison of ENO1/p-ENO1, PI3K/p-PI3K, AKT/p-AKT protein level of A549 cell lines using rs140618127[G]/[A] overexpression plasmid transfection which were compared by t-test.

**Additional file 5 Fig. S5.** Comparison of ENO1/p-ENO1, PI3K/p-PI3K, AKT/p-AKT protein level of PC9 cell lines using rs140618127[G]/[A] overexpression plasmid transfection which were compared by t-test.

**Additional file 6 Fig. S6.** Predicting site of phosphorylation of ENO1. (A) predicting phosphorylation site of ENO1(ENO1 chain A) using NetPhos 3.1; (B) predicting phosphorylation site of ENO1(ENO1 chain A) using Phospho.ELM BLAST; (C) The predicting phosphorylation site of ENO1 chain A using data of PDB database.

**Additional file 7 Fig. S7.** Comparison of PCNA and NH-kB protein level of using rs140618127[G]/[A] overexpression plasmid transfection which were compared by t-test: (A) results of A549 cell lines; (B) result of PC9 cell lines.

**Additional file 8 Fig. S8.** Comparison of β-Catenin, Vimentin, N-Cadherin, E-Cadherin protein level using rs140618127[G]/[A] overexpression plasmid transfection which were compared by t-test: (A) results of A549 cell lines; (B) result of PC9 cell lines.

**Additional file 9 Fig. S9.** Comparison of the H&E staining of p-PI3K, p-Akt, TWIST, N-Cadhersin and SNAIL which were compared by t-test.

**Additional file 10.** Distribution of SNP rs140618127 in different cohorts

**Additional file 11 Table S1.** Associations of lung cancer risk and rs140618127 between NSCLC patients and healthy controls (stratification analysis by smoking/gender/age).

## Data Availability

The datasets used and/or analyzed during the current study are available from the corresponding author on reasonable request.

## Abbreviations

SNPs: Single nucleotide polymorphisms
lncRNAs: Long non-coding RNAs
NSCLC: Non-small cell lung cancer
miRNA: MicroRNA
CMU: China Medical University
H&E: Hematoxylin and eosin
SPSS: Statistical Product and Service Solutions
TCGA: The Cancer Genome Atlas
ceRNA: Competing endogenous RNAs

## References

[1] Bray F, Ferlay J, Soerjomataram I, Siegel RL, Torre LA, Jemal A (2018). Global cancer statistics 2018: GLOBOCAN estimates of incidence and mortality worldwide for 36 cancers in 185 countries. CA Cancer J Clin.

[2] Gutschner T, Hämmerle M, Diederichs S (2013). MALAT1—a paradigm for long noncoding RNA function in cancer. J Mol Med.

[3] Feng N, Ching T, Wang Y, Liu B, Lin H, Shi O (2016). Analysis of microarray data on gene expression and methylation to identify long non-coding RNAs in non-small cell lung Cancer. Sci Rep.

[4] Zhang DD, Wang WT, Xiong J, Xie XM, Cui SS, Zhao ZG (2017). Long noncoding RNA LINC00305 promotes inflammation by activating the AHRR-NF-κB pathway in human monocytes. Sci Rep.

[5] Li S, Shuch BM, Gerstein MB (2017). Whole-genome analysis of papillary kidney cancer finds significant noncoding alterations. PLoS Genet.

[6] Plassais J, Lagoutte L, Correard S, Paradis M, Guaguère E, Hédan B (2016). A point mutation in a lincRNA upstream of GDNF is associated to a canine insensitivity to pain: a spontaneous model for human sensory neuropathies. PLoS Genet.

[7] Verhaegh GW, Verkleij L, Vermeulen SHHM, Heijer MD, Witjes JA, Kiemeney LA. Polymorphisms in the H19 Gene and the Risk of Bladder Cancer: Humana Press; 2008. págs. 710–6 p. 10.1016/j.eururo.2008.01.060 18262338

[8] Jing G, Wei L, Jiayou Z, Xiaoping M, An-Yuan G. lncRNASNP: a database of SNPs in lncRNAs and their potential functions in human and mouse. Nucleic Acids Research. 2015;43(Database issue):D181. 10.1093/nar/gku1000 PMC4383871 25332392

[9] Shiraishi K, Kunitoh H, Daigo Y, Takahashi A, Goto K, Sakamoto H (2012). A genome-wide association study identifies two new susceptibility loci for lung adenocarcinoma in the Japanese population. Nat Genet.

[10] Wei JS, Matsuo K, Chao AH, Shiraishi K, Song M, Kim HN (2017). Association between GWAS-identified lung adenocarcinoma susceptibility loci and EGFR mutations in never-smoking Asian women, and comparison with findings from Western populations. Hum Mol Genet.

[11] Liu Y, Tao Z, Qu J, Zhou X, Zhang C (2017). Long non-coding RNA PCAT7 regulates ELF2 signaling through inhibition of miR-134-5p in nasopharyngeal carcinoma. Biochem Biophys Res Commun.

[12] Chang G-C, Liu K-J, Hsieh C-L, Hu T-S, Charoenfuprasert S, Liu H-K (2006). Identification of α-enolase as an autoantigen in lung cancer: its overexpression is associated with clinical outcomes. Clin Cancer Res.

[13] Fu Q-F, Liu Y, Fan Y, Hua S-N, Qu H-Y, Dong S-W (2015). Alpha-enolase promotes cell glycolysis, growth, migration, and invasion in non-small cell lung cancer through FAK-mediated PI3K/AKT pathway. J Hematol Oncol.

[14] Gong J, Tian J, Lou J, Ke J, Li L, Li J (2016). A functional polymorphism in lnc-LAMC2-1: 1 confers risk of colorectal cancer by affecting miRNA binding. Carcinogenesis..

[15] Miao Y-R, Liu W, Zhang Q, Guo A-Y (2018). lncRNASNP2: an updated database of functional SNPs and mutations in human and mouse lncRNAs. Nucleic Acids Res.

[16] Blom N, Sicheritz-Pontén T, Gupta R, Gammeltoft S, Brunak S (2004). Prediction of post-translational glycosylation and phosphorylation of proteins from the amino acid sequence. Proteomics..

[17] Dinkel H, Chica C, Via A, Gould CM, Jensen LJ, Gibson TJ, et al. Phospho. ELM: a database of phosphorylation sites—update 2011. Nucleic acids research. 2010;39(suppl_1):D261-D7. 10.1093/nar/gkq1104 PMC3013696 21062810

[18] Burley SK, Berman HM, Kleywegt GJ, Markley JL, Nakamura H, Velankar S (2017). Protein data Bank (PDB): the single global macromolecular structure archive.

[19] Do R, Stitziel NO, Won HH, Jørgensen AB, Duga S, Merlini PA (2015). Multiple rare alleles at LDLR and APOA5 confer risk for early-onset myocardial infarction. Nature..

[20] Huyghe JR, Jackson AU, Fogarty MP, Buchkovich ML, Stančáková A, Stringham HM (2012). Exome array analysis identifies new loci and low-frequency variants influencing insulin processing and secretion. Nat Genet.

[21] Ingle JN, Xie F, Ellis MJ, Goss PE, Shepherd LE, Chapman JW (2016). Genetic polymorphisms in the long noncoding RNA MIR2052HG offer a Pharmacogenomic basis for the response of breast Cancer patients to aromatase inhibitor therapy. Cancer Res.

[22] Tang X, Gao Y, Yu L, Lu Y, Zhou G, Cheng L (2017). Correlations between lncRNA-SOX2OT polymorphism and susceptibility to breast cancer in a Chinese population. Biomark Med.

[23] Ling H, Spizzo R, Atlasi Y, Nicoloso M, Shimizu M, Redis RS (2013). CCAT2, a novel noncoding RNA mapping to 8q24, underlies metastatic progression and chromosomal instability in colon cancer. Genome Res.

[24] Russell MR, Penikis A, Oldridge DA, Alvarez-Dominguez JR, McDaniel L, Diamond M (2015). CASC15-S is a tumor suppressor lncRNA at the 6p22 neuroblastoma susceptibility locus. Cancer Res.

[25] Wang Y, He H, Li W, Phay J, Shen R, Yu L, et al. MYH9 binds to lncRNA gene PTCSC2 and regulates FOXE1 in the 9q22 thyroid cancer risk locus. Proceedings of the National Academy of Sciences of the United States of America. 2017;114(3). 10.1073/pnas.1619917114 PMC5255605 28049826

[26] Su F, Li D, Zeng M, Song E (2015). A cytoplasmic NF-kB interacting long noncoding RNA blocks IkB phosphorylation and suppresses breast cancer metastasis. Cancer Cell.

[27] Tang J, Zhong G, Zhang H, Yu B, Wei F, Luo L. LncRNA DANCR upregulates PI3K/AKT signaling through activating serine phosphorylation of RXRA. Cell death & disease. 2018;9(12). 10.1038/s41419-018-1220-7 PMC6281578 30518934

[28] Wang H, Liu M, Fang L, Jiang J, Zhang Z, Kuang Y (2018). The cisplatin-induced lncRNA PANDAR dictates the chemoresistance of ovarian cancer via regulating SFRS2-mediated p53 phosphorylation. Cell Death Dis.

[29] Jiang Y, Lin L, Zhong S, Cai Y, Zhang F, Wang X, et al. Overexpression of novel lncRNA NLIPMT inhibits metastasis by reducing phosphorylated glycogen synthase kinase 3β in breast cancer. J Cell Physiol. 2018. 10.1002/jcp.27738 30417392

[30] Yang Y, Jiang C, Yang Y, Guo L, Huang J, Liu X (2018). Silencing of LncRNA-HOTAIR decreases drug resistance of non-small cell lung Cancer cells by inactivating autophagy via suppressing the phosphorylation of ULK1. Biochem Biophys Res Commun.

[31] Zeng S, Xie X, Xiao Y-F, Tang B, Hu C-J, Wang S-M (2018). Long noncoding RNA LINC00675 enhances phosphorylation of vimentin on Ser83 to suppress gastric cancer progression. Cancer Lett.

[32] Ning S, Yue M, Wang P, Liu Y, Zhi H, Zhang Y (2016). LincSNP 2.0: an updated database for linking disease-associated SNPs to human long non-coding RNAs and their TFBSs. Nucleic Acids Res.

[33] Gao X, Li S, Li W, Wang G, Zhao W, Han J, et al. MicroRNA-539 suppresses tumor cell growth by targeting the WNT8B gene in non-small cell lung cancer. J Cell Biochem. 2018. 10.1002/jcb.26634 29266418

[34] Guo J, Gong G, Zhang B. miR-539 acts as a tumor suppressor by targeting epidermal growth factor receptor in breast cancer. Scientific Reports. 2018;8(1):2073. 10.1038/s41598-018-20431-z PMC5794864 29391441

[35] Cappello P, Principe M, Bulfamante S, Novelli F. Alpha-Enolase (ENO1), a potential target in novel immunotherapies. Frontiers in bioscience (Landmark edition). 2017;22:944–59. 10.2741/4526 27814656

