# Supplementary figures and images for: A SNP-mediated lncRNA (LOC146880) and microRNA (miR-539-5p) interaction and its potential impact on the NSCLC risk
Source: J Exp Clin Cancer Res. 2020 Aug 14;39:157. doi: 10.1186/s13046-020-01652-5 (PMC7427888; doi:10.1186/s13046-020-01652-5)

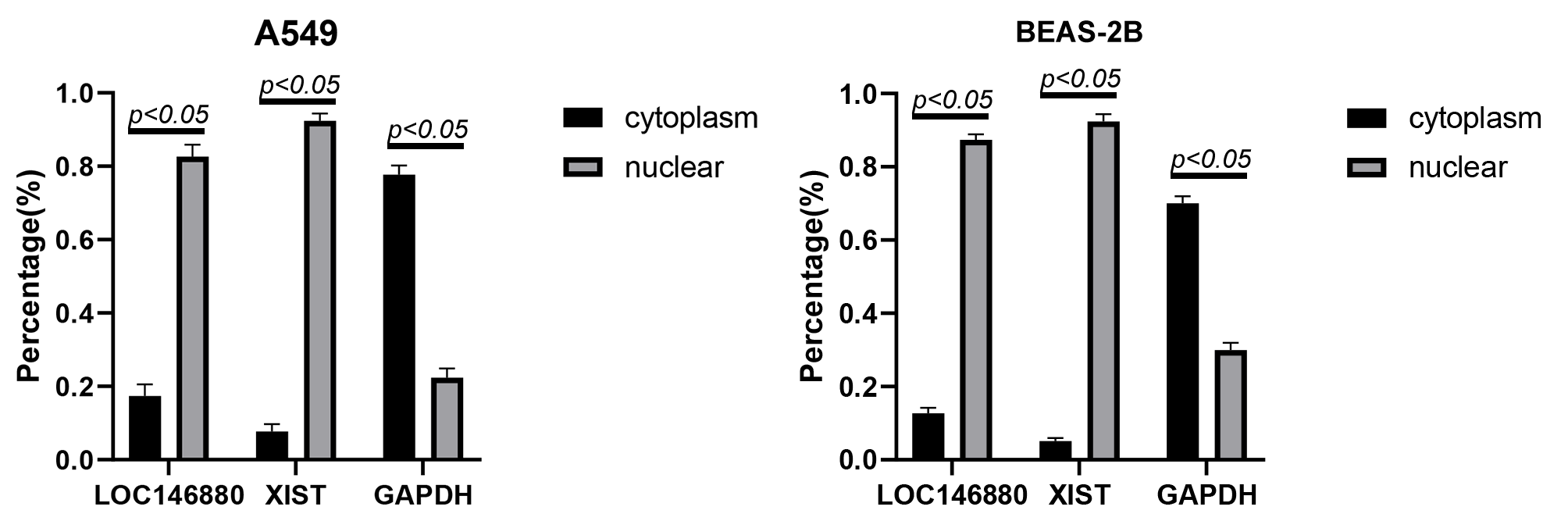

Supplement: Supplementary file 1 — Additional file 1 Fig. S1. Locations of LOC146880 (A549 & BEAS2B): LOC146880 locals mainly in cytoplasm. The comparison between two groups using t-test. [file 13046_2020_1652_MOESM1_ESM.tif]

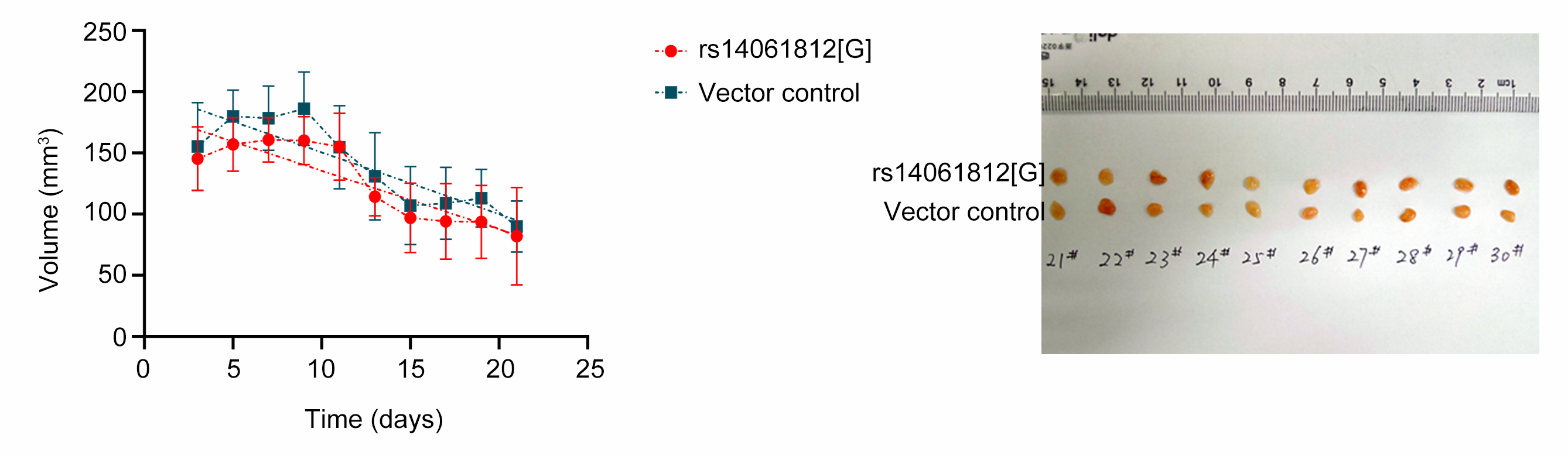

Supplement: Supplementary file 2 — Additional file 2 Fig. S2. Comparison of tumor size between vector control group and the wide type: There was no significant difference of tumor size between vector control group and the wide type, rs14061812[G] [file 13046_2020_1652_MOESM2_ESM.tif]

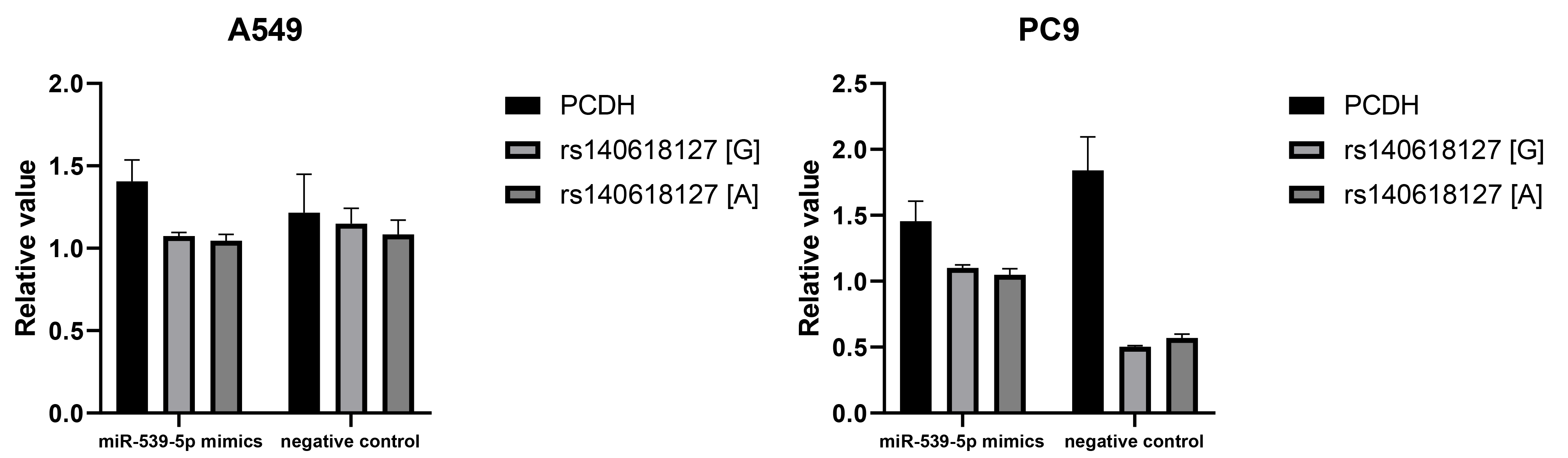

Supplement: Supplementary file 3 — Additional file 3 Fig. S3. Comparison of ENO1 protein level of rs140618127[G]/[A] by overexpression plasmid transfection which were compared by t-test. [file 13046_2020_1652_MOESM3_ESM.tif]

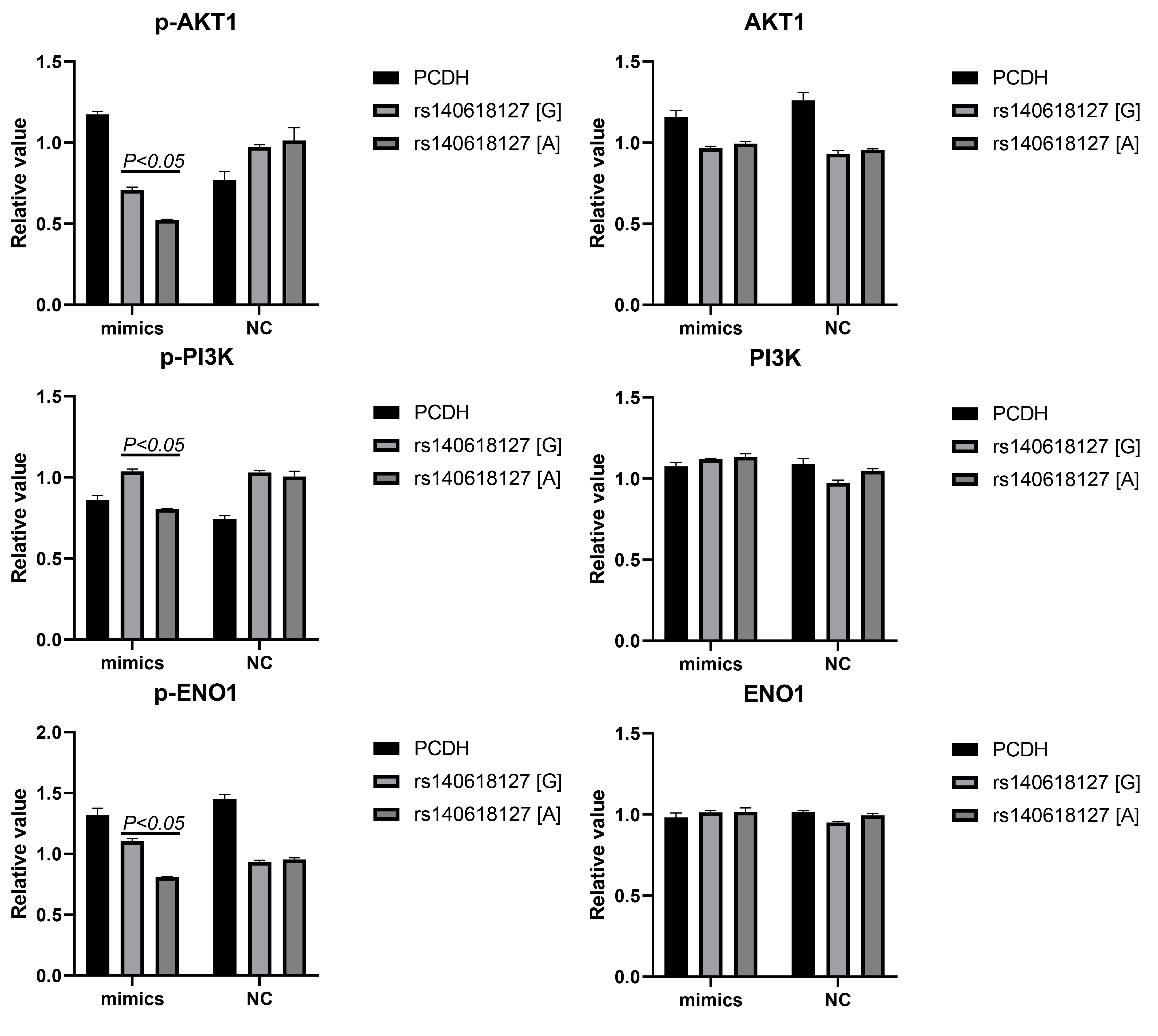

Supplement: Supplementary file 4 — Additional file 4 Fig. S4. Comparison of ENO1/p-ENO1, PI3K/p-PI3K, AKT/p-AKT protein level of A549 cell lines using rs140618127[G]/[A] overexpression plasmid transfection which were compared by t-test. [file 13046_2020_1652_MOESM4_ESM.tif]

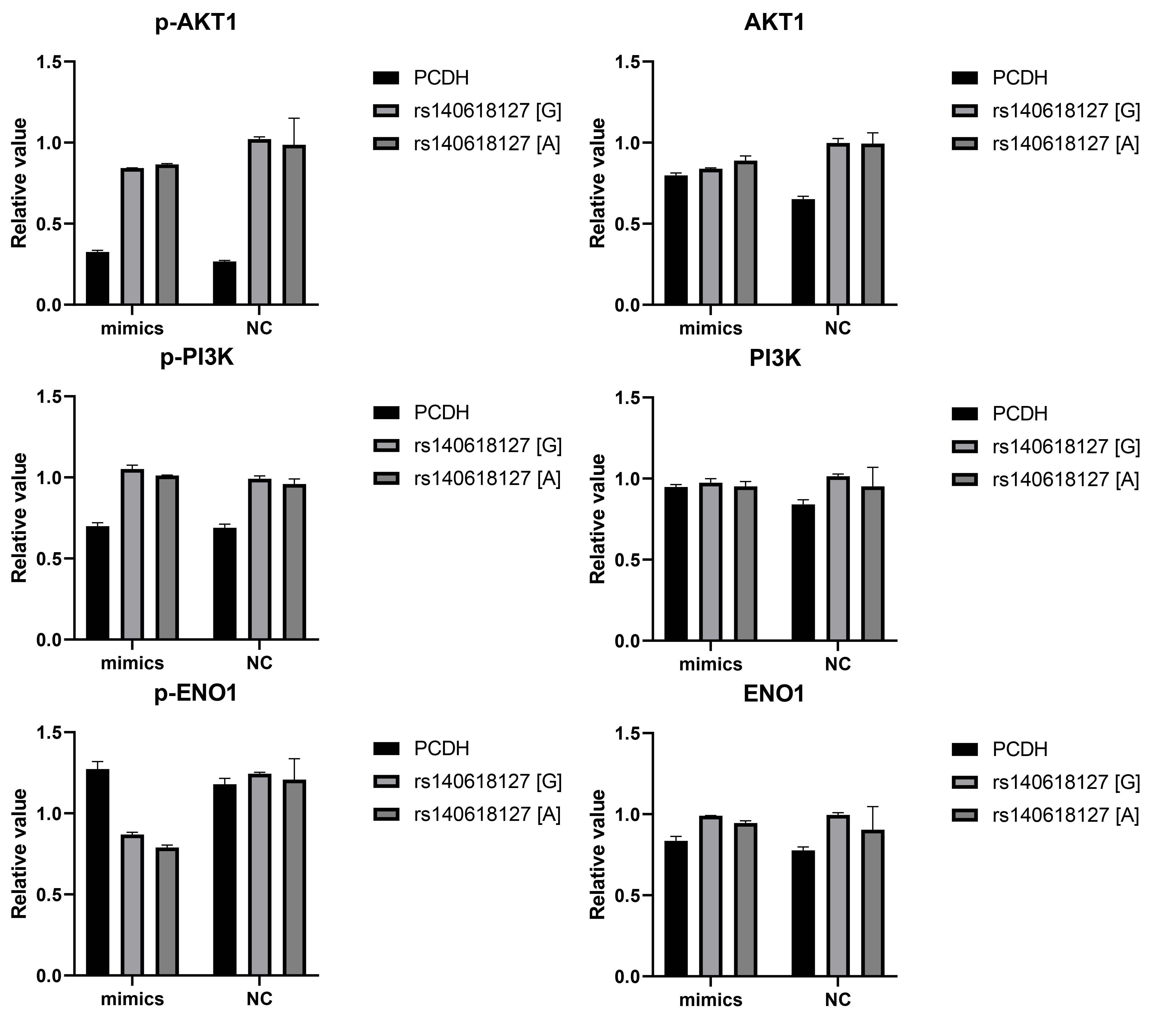

Supplement: Supplementary file 5 — Additional file 5 Fig. S5. Comparison of ENO1/p-ENO1, PI3K/p-PI3K, AKT/p-AKT protein level of PC9 cell lines using rs140618127[G]/[A] overexpression plasmid transfection which were compared by t-test. [file 13046_2020_1652_MOESM5_ESM.tif]

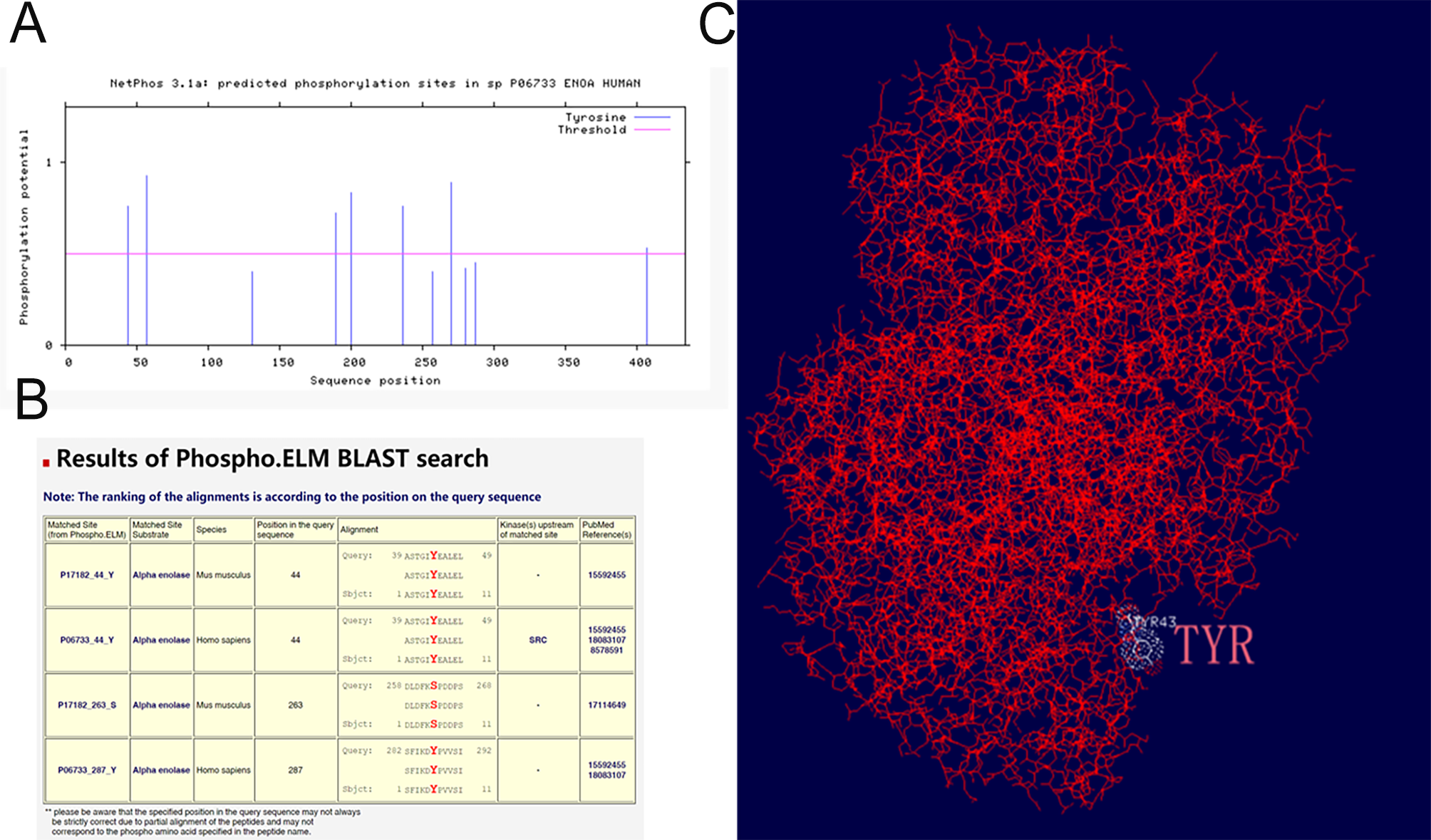

Supplement: Supplementary file 6 — Additional file 6 Fig. S6. Predicting site of phosphorylation of ENO1. (A) predicting phosphorylation site of ENO1(ENO1 chain A) using NetPhos 3.1; (B) predicting phosphorylation site of ENO1(ENO1 chain A) using Phospho.ELM BLAST; (C) The predicting phosphorylation site of ENO1 chain A using data of PDB database. [file 13046_2020_1652_MOESM6_ESM.tif]

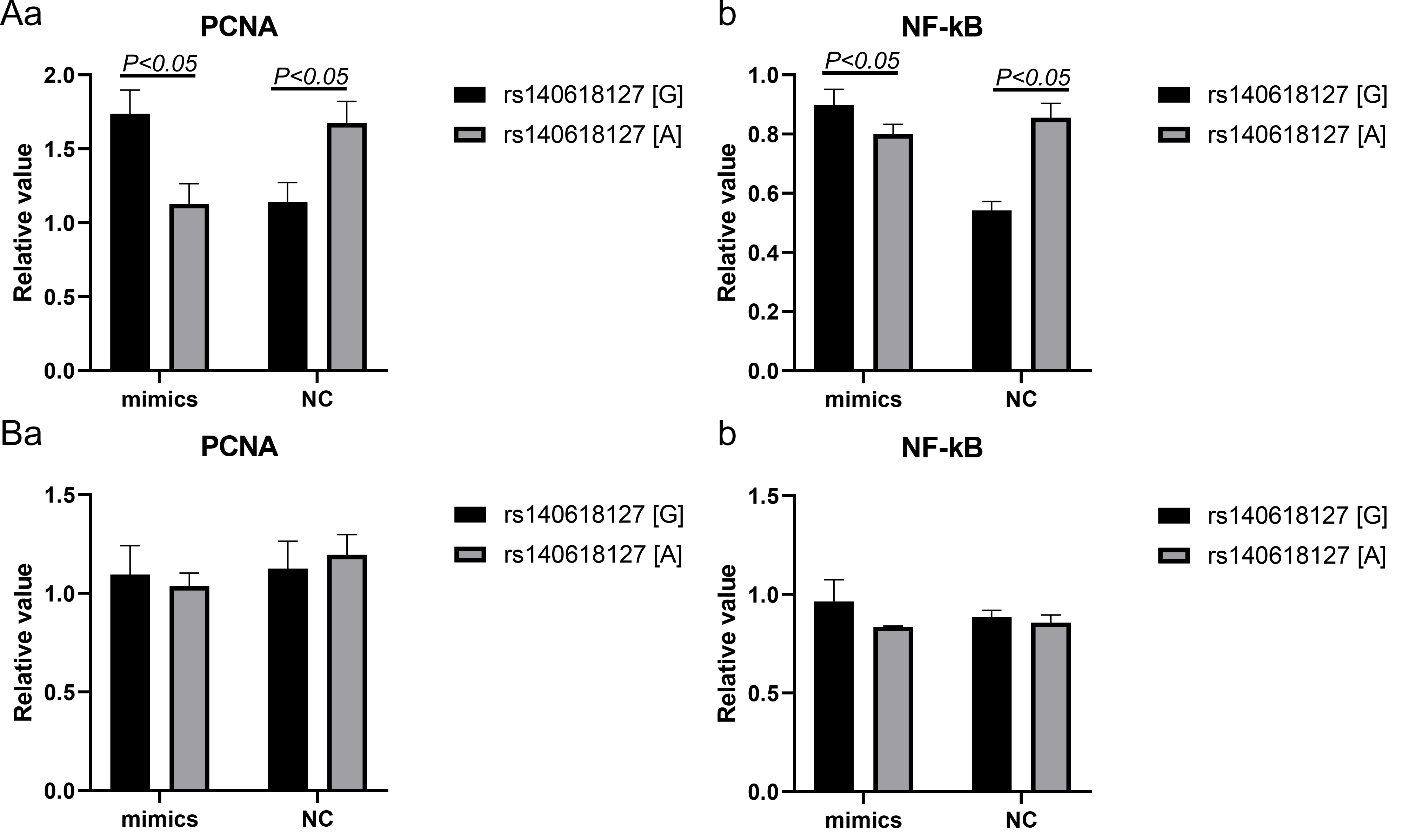

Supplement: Supplementary file 7 — Additional file 7 Fig. S7. Comparison of PCNA and NH-kB protein level of using rs140618127[G]/[A] overexpression plasmid transfection which were compared by t-test: (A) results of A549 cell lines; (B) result of PC9 cell lines. [file 13046_2020_1652_MOESM7_ESM.tif]

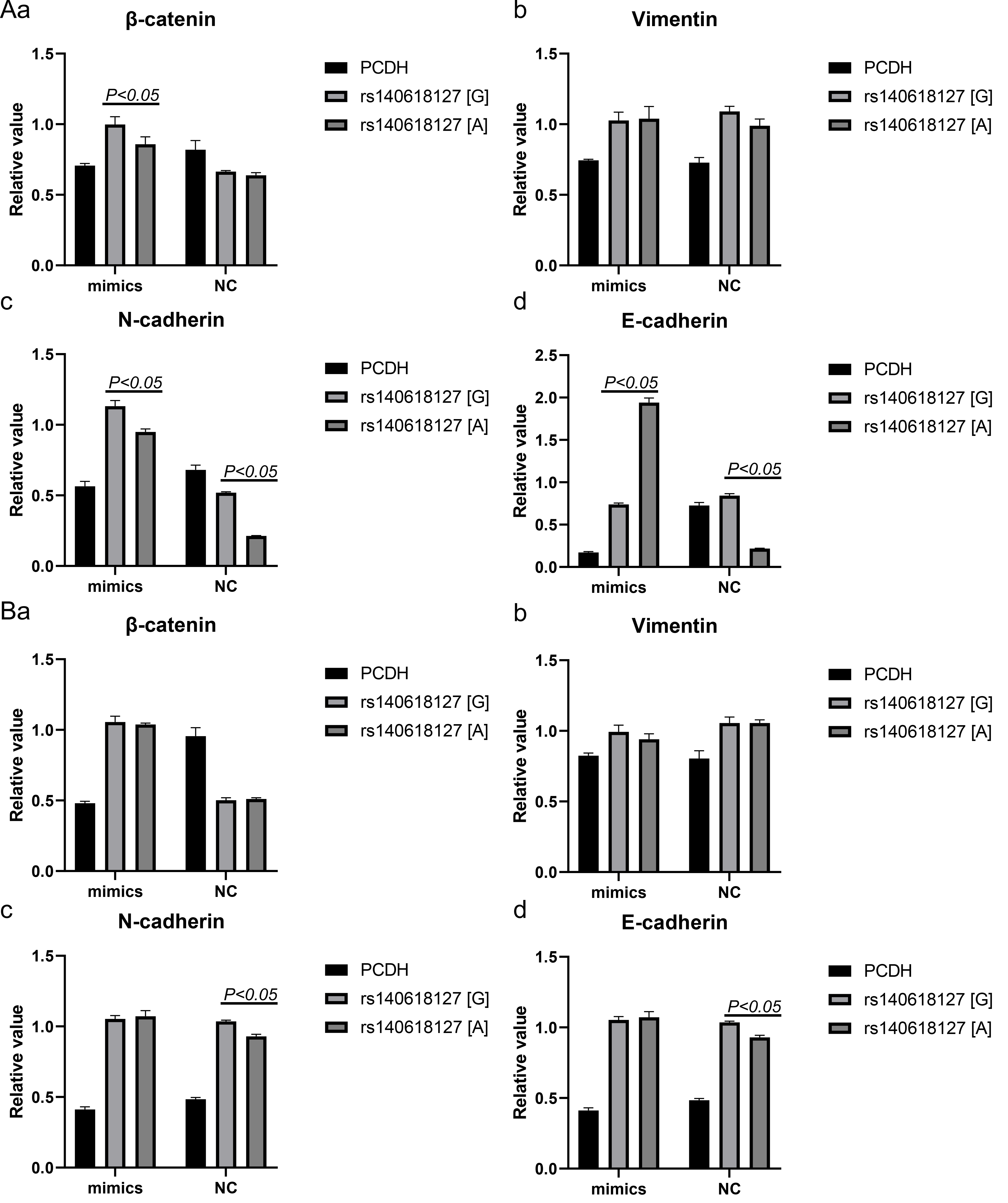

Supplement: Supplementary file 8 — Additional file 8 Fig. S8. Comparison of β-Catenin, Vimentin, N-Cadherin, E-Cadherin protein level using rs140618127[G]/[A] overexpression plasmid transfection which were compared by t-test: (A) results of A549 cell lines; (B) result of PC9 cell lines. [file 13046_2020_1652_MOESM8_ESM.tif]

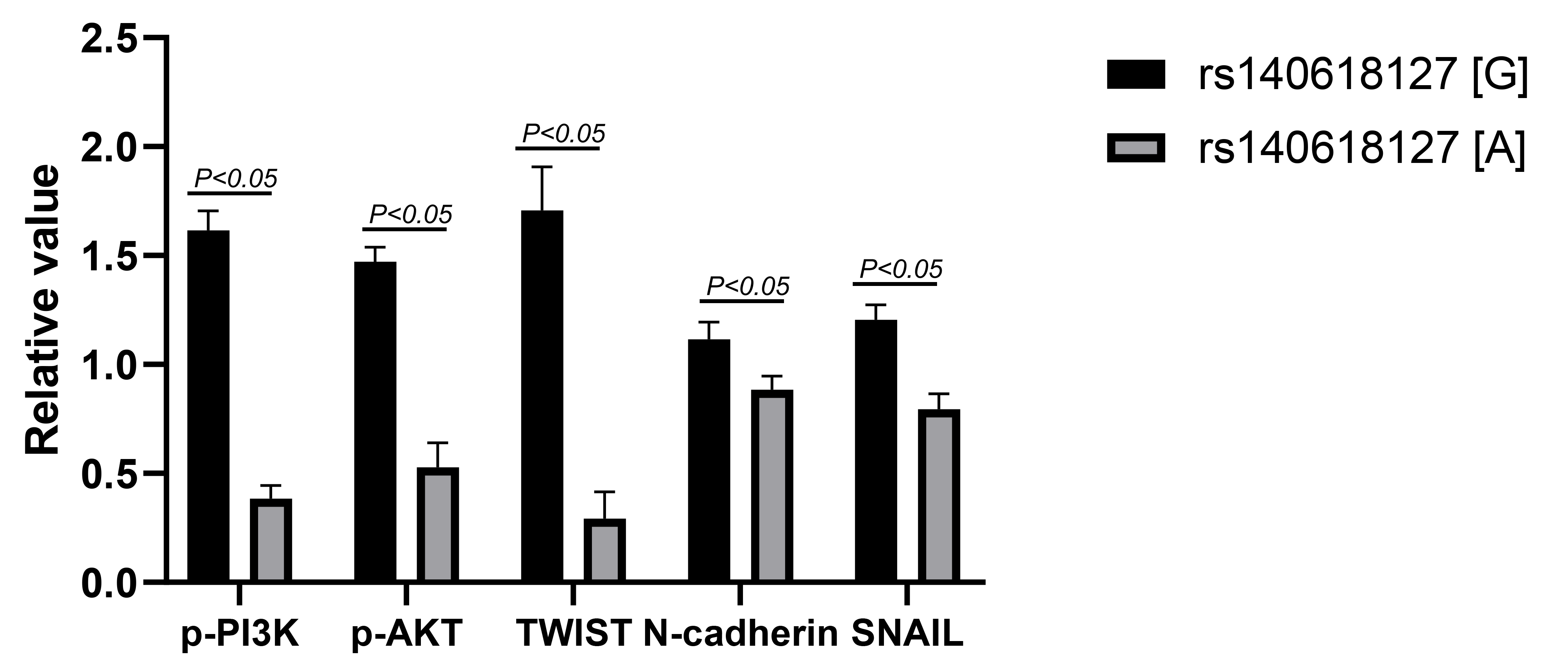

Supplement: Supplementary file 9 — Additional file 9 Fig. S9. Comparison of the H&E staining of p-PI3K, p-Akt, TWIST, N-Cadhersin and SNAIL which were compared by t-test. [file 13046_2020_1652_MOESM9_ESM.tif]
