# Supplementary material for: A SNP-mediated lncRNA (LOC146880) and microRNA (miR-539-5p) interaction and its potential impact on the NSCLC risk
Source: J Exp Clin Cancer Res. 2020 Aug 14;39:157. doi: 10.1186/s13046-020-01652-5 (PMC7427888; doi:10.1186/s13046-020-01652-5)
Supplement: Supplementary file 11 — Additional file 11 Table S1. Associations of lung cancer risk and rs140618127 between NSCLC patients and healthy controls (stratification analysis by smoking/gender/age). [file 13046_2020_1652_MOESM11_ESM.docx]

**Table S1 Associations of lung cancer risk and rs140618127 between NSCLC patients and healthy controls (stratification analysis by smoking/gender/age)**

| Variables | control（%） | | NSCLC（%） | | P value | OR value^*^ |
| --- | --- | --- | --- | --- | --- | --- |
|  | G allele | A allele | G allele | A allele |  |  |
| Age at diagnosis (years) |  |  |  |  |  |  |
| <60 | 1179(98.00%) | 24(2.00%) | 599(99.17%) | 5(0.83%) | 0.071 | 0.41 |
| ≥60 | 297(97.06%) | 9(2.94%) | 589(99.16%) | 5(0.84%) | 0.024 | 0.28 |
| Gender |  |  |  |  |  |  |
| male | 414(98.10%) | 8(1.90%) | 540(99.26%) | 4(0.74%) | 0.119 | 0.38 |
| female | 1062(97.70%) | 25(2.30%) | 648(99.08%) | 6(0.92%) | 0.041 | 0.39 |
| Smoking status |  |  |  |  |  |  |
| no | 1262(97.60%) | 31(2.40%) | 659(99.10%) | 6(0.90%) | 0.027 | 0.37 |
| yes | 214(99.07%) | 2(0.93%) | 527(99.25%) | 4(0.75%) | 0.811 | 0.81 |

^*^The reference is G allele.
